# Identification and Functional Insights of Knickkopf Genes in the Larval Cuticle of *Leptinotarsa decemlineata*

**DOI:** 10.3390/insects15080623

**Published:** 2024-08-19

**Authors:** Mu-Zi Zeng, Wei Zhou, Shan-Shan Wen, Hao Wu, Qing Zhang, Kai-Yun Fu, Wen-Chao Guo, Ji-Feng Shi

**Affiliations:** 1 State Key Laboratory of Resource Insects, College of Sericulture, Textile, and Biomass Sciences, Southwest University, Chongqing 400715, China; mz_zeng@163.com (M.-Z.Z.);; 2 Institute of Plant Protection Xinjiang Academy of Agricultural Sciences/Key Laboratory of Integrated Pest Management on Crops in Northwestern Oasis, Ministry of Agriculture/Xinjiang Key Laboratory of Agricultural Biosafety, Urumqi 830091, China; fukaiyun000@foxmail.com (K.-Y.F.); gwc1966@163.com (W.-C.G.)

**Keywords:** Knickkopf, larval cuticle, RNA interference, *Leptinotarsa decemlineata*

## Abstract

**Simple Summary:**

The Colorado potato beetle, *Leptinotarsa decemlineata*, poses a significant threat to potato crops worldwide, causing substantial agricultural damage. To better understand how these pests develop and potentially find ways to control them, we studied a group of genes called *Knickkopf* (*Knk*) genes in the larvae of the beetle. These genes are crucial for forming and maintaining the insect’s outer shell, known as the cuticle, which protects them from the environment. We identified four *LdKnk*-family genes in the beetle and analyzed when and where these genes are expressed during the insect’s growth stages. By using RNA interference (RNAi) to silence these genes, we observed that larvae became weaker, with damaged cuticles and higher death rates. These findings indicate that *LdKnk*-family genes are vital for the beetle’s survival and development. Targeting *LdKnk*-family genes could provide a new strategy for pest control, potentially weakening the beetles’ defenses and making them more susceptible to environmental factors and insecticides, ultimately helping to protect potato crops.

**Abstract:**

The Colorado potato beetle (*Leptinotarsa decemlineata*) is a major pest of potato crops. While *Knickkopf* (*Knk*) genes are essential for insect cuticle formation, their roles in pests like *L. decemlineata* remain unclear. This study aims to identify and characterize *Knk* genes in *L. decemlineata* and explore their functions in larval development and cuticle integrity. We used genomic and transcriptomic databases to identify *LdKnk*-family genes, validated through RT-PCR and RACE. Gene expression was analyzed at various developmental stages and tissues using qRT-PCR. RNA interference (RNAi) and Transmission electron microscopy (TEM) were applied to determine the functional roles of these genes. Four *LdKnk*-family genes were identified. Spatio-temporal expression analysis indicated significant gene expression during larval molting and pupal stages, especially in the epidermis. RNAi experiments showed that silencing *LdKnk* and *LdKnk3-5*′ led to reduced larval weight, cuticle thinning, and increased mortality, while *LdKnk3-FL* knockdown caused abnormal cuticle thickening and molting disruptions. *LdKnk2* knockdown increased epicuticle and endocuticle thickness without visible phenotypic changes. The study highlights the essential roles of *LdKnk*-family genes in maintaining cuticle structure and integrity, suggesting their potential as targets for RNAi-based pest control.

## 1. Introduction

The insect cuticle is essential for maintaining an insect’s shape and providing protection against dehydration, pathogens, and environmental damage [1,2]. To accommodate growth, insects periodically shed their cuticle through molting, replacing the old cuticle with a new one [3,4,5,6]. TEM has been crucial for studying the ultrastructure of the insect cuticle, revealing its multi-layered architecture and the organization of chitin microfibrils. TEM studies have shown that the cuticle consists of three layers forming an extracellular matrix. The outer waxy envelope (env) acts as the primary barrier against water loss and foreign substances. Beneath it, the protein-rich epicuticle (epi) contributes to the insect’s overall body shape. The innermost procuticle (pro), composed of a protein-chitin matrix, provides stability [7,8,9].

Chitin, a homopolymer of β-1,4-linked N-acetylglucosamine units, is the main structural component of the insect cuticle [10,11,12,13,14,15]. When associated with proteins, chitin arranges into microfibrils that align parallel to each other, forming stacked helicoidal laminae [16,17,18,19]. Cuticle-associated proteins significantly influence chitin organization [1,20,21]. Among these, the chitin-binding protein Knickkopf (Knk) has been extensively studied in *Drosophila melanogaster*, *Tribolium castaneum,* and *Locusta migratoria* [22]. In *D. melanogaster*, Knk is crucial for chitin organization within the tracheal system, the larval body, and the adult wing cuticle [23,24]. Knk proteins comprise a signal peptide, two DM13 domains of unknown function, and a dopamine-monooxygenase (DOMON) domain essential for its activity [25]. It interacts with the chitin-binding protein Obstructor-A (Obst-A) and the chitin deacetylase Serpentine (Serp) to form a complex at the apical membrane, facilitating the deposition and organization of chitin fibers [26,27].

Studies on *T. castaneum* have revealed that Knk proteins bind to chitin, protecting it from enzymatic degradation during molting and ensuring proper laminar organization, playing roles in cuticle formation at different developmental stages and in different parts of the insect anatomy [28,29,30]. Additionally, in *L. migratoria*, *Lm*Knk contributes to correct chitin content, pore canal formation, and lipid deposition during molting, with *Lm*Knk3-5′ being essential for cuticle formation [31,32]. These findings suggest that *Knk*-family proteins may exhibit functional diversity across different insect species.

The Colorado potato beetle, *L. decemlineata*, is a significant agricultural pest that poses a severe threat to potato crops and farmers’ livelihoods [33,34]. Although insecticides have historically been effective in suppressing *L. decemlineata* populations, the emergence of insecticide-resistant populations has created significant management challenges [35,36,37]. Understanding the genetic and molecular mechanisms of cuticle formation in this species could offer valuable insights for pest management.

In this study, we aim to identify and characterize *Knk* genes in *L. decemlineata* and investigate their roles in larval development and cuticle integrity. We identified four *Knk*-family genes: *LdKnk*, *LdKnk2*, *LdKnk3-full length* (*LdKnk3-FL*), and *LdKnk3-5*′. Using qRT-PCR, we analyzed their expression across various developmental stages and tissues. RNAi experiments assessed their impact on larval development and cuticle structure. Our results demonstrate that *Knk*-family genes exhibit developmentally regulated and tissue-specific expression patterns. Specifically, *LdKnk*, *LdKnk3-FL*, and *LdKnk3-5*′ are essential for larval development in *L. decemlineata*, as confirmed by TEM analysis. These findings highlight potential targets for pest management and contribute to understanding the function of *Knk* genes in insects.

## 2. Materials and Methods

### 2.1. Experimental Insect

The rearing of *L. decemlineata* followed standardized protocols as previously described [38]. Adults were collected from potato fields in Urumqi, Xinjiang (43.82° N, 87.61° E) during their spring emergence. The beetles were kept at 28 ± 1 °C with a 14:10 light-dark cycle and 50–60% humidity and were fed fresh potato leaves. Eggs were laid on the underside of potato leaves and hatched into larvae within seven days. The larvae underwent four instars, with the first three instars, each lasting two days and the fourth instar lasting 3.5 days. After the larval period, the fourth-instar larvae ceased feeding, burrowed into the soil for pupation, and subsequently emerged as adults.

### 2.2. Identification of LdKnk-Family Genes

Using the known amino acid sequences of Knk proteins from *T. castaneum*, *D. melanogaster*, and *L. migratoria* as queries, a genome-wide TBLASTN search was conducted on the *L. decemlineata* genome data (https://www.hgsc.bcm.edu/arthropods/colorado-potato-beetle-genome-project (accessed on 1 January 2024), PRJNA854273). This search resulted in the identification of four putative *Knk-like* genes in *L. decemlineata*. These sequences were further used to identify additional *Knk-like* genes from the nonredundant (nr) NCBI nucleotide database and transcriptome databases (PRJNA464380 and our unpublished database) of *L. decemlineata*.

The sequences were verified via polymerase chain reaction (PCR) using primers listed in Table S1. They were then completed by 5′- and 3′-RACE using the SMARTer RACE cDNA amplification kit (Takara Bio., Dalian, China). Table S1 provides the gene-specific and nested primers for the 5′ and 3′ ends. The full-length *Knk*-family gene sequences have been submitted to GenBank, with Accession numbers listed in Table 1.

### 2.3. Bioinformatics and Phylogenetic Analysis

Knk proteins open reading frames (ORFs) and amino acid sequences were identified, and their theoretical isoelectric points and molecular weights were calculated using ExPASy’s online server (https://web.expasy.org/peptide_mass/, accessed on 1 January 2024). The results are detailed in Table 1. The exon-intron organization was predicted by aligning ORFs with their corresponding genomic sequences, and these organizations were illustrated using Adobe Illustrator CS6. Domain analysis was performed using SMART software (http://smart.embl-heidelberg.de/ (accessed on 1 January 2024)).

To investigate evolutionary relationships, a phylogenetic tree based on Knk amino acid sequences from ten insect species was constructed using MEGA6 software. The unrooted phylogenetic tree was generated using the neighbor-joining method and the Jones-Taylor-Thornton (JTT) substitution model, based on full-length protein sequence alignments. Bootstrap analysis with 1000 replications was performed, and bootstrap values greater than 50% are indicated on the tree. Sequence accession numbers for Knk genes from various insects are provided in Table S2.

### 2.4. Expression Analysis of LdKnk-Family Genes

Quantitative real-time PCR (qRT-PCR) was employed to evaluate the temporal and spatial expression patterns of *LdKnk*-family genes. Gene-specific primers (Table S1) were designed using the PRIMER PREMIER 5 program. cDNA templates were generated from different developmental stages and tissues.

For developmental stage expression analysis, cDNA templates were obtained from eggs, first- to third-instar larvae, wandering larvae, prepupae, and pupae at one-day intervals, and from fourth-instar larvae at twelve-hour intervals. For tissue-specific expression analysis, RNA samples were extracted from the epidermis, foregut, midgut, hindgut, Malpighian tubules, fat body, hemocytes, and trachea of day-3 fourth-instar larvae. Each sample consisted of RNA from 5–30 individuals and was processed in triplicate.

RNA extraction was performed using the SV Total RNA Isolation System (Promega), followed by DNase I treatment to remove residual DNA. mRNA expression levels were quantified using the ABI 7500 Real-Time PCR System (Applied Biosystems, Foster City, CA, USA) and the TaKaRa SYBR Premix Ex Taq kit, following a standard PCR protocol. Internal control genes (Table S1) were used for normalization as per our published results [39]. RT negative controls (without reverse transcriptase) and non-template negative controls were included to ensure the absence of genomic DNA and contamination. Each sample was tested in triplicate. Data analysis was performed using the 2^−ΔΔCT^ method, normalized to the geometric mean of the internal control genes, and validated according to the MIQE guidelines [40].

### 2.5. Preparation of dsRNA Using Bacterial Expression

To enhance the reliability of RNAi experiments, two different dsRNA fragments were designed for each *LdKnk*-family gene. The dsRNAs were prepared as described previously [41]. Unique regions of each gene were amplified using gene-specific primers (Table S1) and introduced into a T7 dual promoter expression system in *Escherichia coli* HT115 (DE3) cells, which lack RNase III, to produce dsRNA. As a control, dsRNA targeting the enhanced green fluorescent protein (EGFP) gene from *Aequorea victoria* was produced to monitor the nonspecific effects of dsRNA.

Bacterial cultures were inoculated and grown to an OD_600_ of 1.0. dsRNA expression and were then induced by adding isopropyl β-D-1-thiogalactopyranoside (IPTG) to a final concentration of 0.1 mM. The dsRNA was extracted and confirmed by electrophoresis on a 1% agarose gel. Post-verification, the bacteria were harvested by centrifugation at 5000× *g* for 10 min and resuspended in 0.05 M phosphate-buffered saline (PBS, pH 7.4) at a 1:1 ratio for bioassays. The final concentration of dsRNA in the bacterial suspension was approximately 0.5 g/mL.

### 2.6. Dietary dsRNA Bioassays

Bioassays were conducted as previously described [14,15]. Newly ecdysed second-instar larvae were selected to assess the impact of dsRNA ingestion. Ten larvae per treatment group were fed leaves immersed in specific dsRNA targeting *LdKnk*-family genes for three days, following a four-hour starvation period. Control groups included a blank control (PBS buffer) and a negative control (ds*GFP*). Each treatment was replicated 15 times.

On the third day, total RNA was extracted from three replicates of each treatment group (10 larvae per replicate) to assess target gene transcript levels using qRT-PCR. The remaining 12 replicates per treatment were provided with untreated leaves under standard conditions for further observation. This included phenotypic analysis, weight measurements on days 3 and 6, and larval survival rates on day 6. Additionally, cuticle structure analysis was performed using TEM, and quantifications of overall procuticle, epicuticle, envcuticle, and layered procuticle thickness were measured using ImageJ software based on larval cuticle TEM images. Each bioassay was performed with three biological replicates to ensure accuracy and reproducibility.

### 2.7. Transmission Electron Microscopy (TEM) Analysis

For TEM analysis, larvae were dissected on the third day of the fourth instar following the *LdKnk*-family genes knockdown to obtain integument samples. The dorsal integument was excised and fixed overnight in 3% glutaraldehyde at 4 °C. Samples were then rinsed three times with PBS buffer (pH 7.2) for 15 min each and postfixed with 1% osmium tetroxide (OsO_4_) solution for 1–2 h. Following postfixation, the samples were rinsed in 0.1 M PBS (pH 7.2) and dehydrated through a graded ethanol series (30%, 50%, 70%, 80%, 95%, and 100%).

The dehydrated samples were embedded in Epon 812 at room temperature for an appropriate duration. The resin blocks were then trimmed to 60–70 nm thickness using an ultramicrotome (Leica UCT, Wetzlar, Germany) and transferred onto copper grids. Sections were stained sequentially with uranyl acetate and lead citrate. After drying, images were captured using a Transmission Electron Microscope (Hitachi HT-7800, Tokyo, Japan) at 80 kV.

### 2.8. Data Analysis

Data from three biological replicates were pooled and presented as means ± SE. Statistical analyses were conducted using One-way ANOVA with the Tukey-Kramer test to determine significant differences (* *p* < 0.05, ** *p* < 0.01, *** *p* < 0.001), utilizing Prism 10 software. No significant differences were found between dsRNAs targeting two distinct regions of each of the four genes: ds*Knk-1* and ds*Knk-2*, ds*Knk2-1* and ds*Knk2-2*, ds*Knk3-FL-1* and ds*Knk3-FL-2*, ds*Knk3-5′-1* and ds*Knk3-5′-2*. Consequently, data from the RNAi treatments for each gene were combined for further analysis.

## 3. Results

### 3.1. Identification and Characterization of LdKnk-Family Genes in L.decemlineata

To identify *Knk* genes in *L. decemlineata*, comprehensive searches were conducted using genomes [42,43] and transcriptome databases [44]. Amino acid sequences of *Knk* genes from *T. castaneum*, *D. melanogaster*, and *L. migratoria* were used as queries in BLAST searches. which resulted in the identification of four candidate gene fragments encoding *Ld*Knk-like proteins.

These gene fragments were validated through reverse-transcription PCR (RT-PCR) followed by sequencing. The application of rapid amplification of cDNA ends (RACE) subsequently identified four full-length *LdKnk* gene sequences in *L. decemlineata*, named *LdKnk*, *LdKnk2*, *LdKnk3-FL*, and *LdKnk3-5*′. The accession numbers are listed in Table 1.

The open reading frames (ORFs) and protein characteristics of these genes are as follows. *LdKnk* comprises 2028 nucleotides encoding a 76.03 kDa protein with 675 amino acids and an isoelectric point (pI) of 6.35. *LdKnk2* comprises 2109 nucleotides encoding a 78.90 kDa protein with 702 amino acids and a pI of 5.09. *LdKnk3-FL* comprises 4188 nucleotides encoding a 156.48 kDa protein with 1395 amino acids and a pI of 7.41. *LdKnk3-5*′ comprises 915 nucleotides encoding a 33.81 kDa protein with 304 amino acids and a pI of 7.65. Additionally, two alternative splicing transcripts of *LdKnk3* were identified. *LdKnk3-FL* encodes a full-length protein, while *LdKnk3-5*′ encodes a C-terminally truncated protein (Table 1).

**Figure 1:**
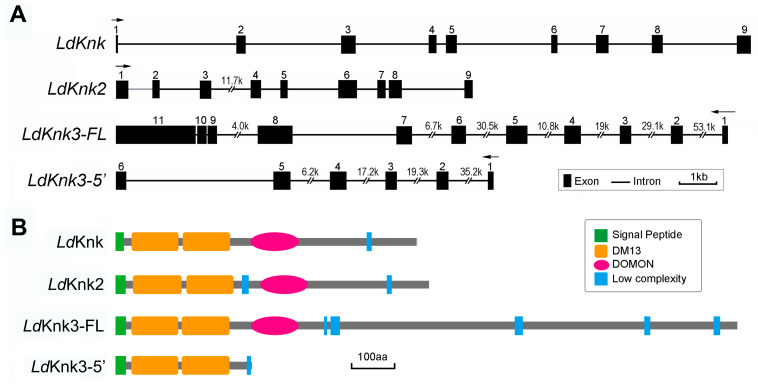
The Exon-intron and Domain architecture of the putative *LdKnk*-family proteins from *L. decemlineata*. (**A**) A schematic diagram of the exon-intron organization of each *LdKnk*-family gene was determined by sequence comparison between the genomic sequence and the Complete cDNA sequence. Black closed boxes mark exons and lines mark introns. Since the first 281 nucleotides of *LdKnk2* cDNA cannot be found in the *L. decemlineata* genome database, we cannot reveal the exact location of the first intron as shown in the grey line. (**B**) Domain architecture of deduced amino acid sequences of *Ld*Knk-family proteins as predicted using SMART protein. The green bar is the signal peptide; the orange bar is the DM13 domain; the rose oval is the dopamine monooxygenase N-terminal-like (DOMON) domain; the pink is the coiled-coil region; and the blue bar is the low complexity region.

Comparing the full-length cDNA sequences with the genomic DNA sequences of *L. decemlineata* allowed us to construct gene structures. Both *LdKnk* and *LdKnk2* contain nine exons, *LdKnk3-FL* contains eleven exons, and *LdKnk3-5*′ contains six exons. Notably, the first 281 nucleotides from the 5′-end of the *LdKnk2* cDNA sequence could not be located in the genome database, preventing the determination of the first intron location (indicated by a grey line). Exons 1–5 of *LdKnk3-5*′ are identical to the 5′ end sequence of *LdKnk3-FL* (Figure 1A).

The domain architectures of these four Knk proteins were predicted using their amino acid sequences and SMART software. All four predicted proteins contain a signal peptide and two N-terminal DM13 domains. Additionally, *LdKnk*, *LdKnk2*, and *LdKnk3-FL* contain a dopamine monnoxygenase (DOMON) domain in the middle, which is absent in *LdKnk3-5*′ (Figure 1B).

**Figure 2:**
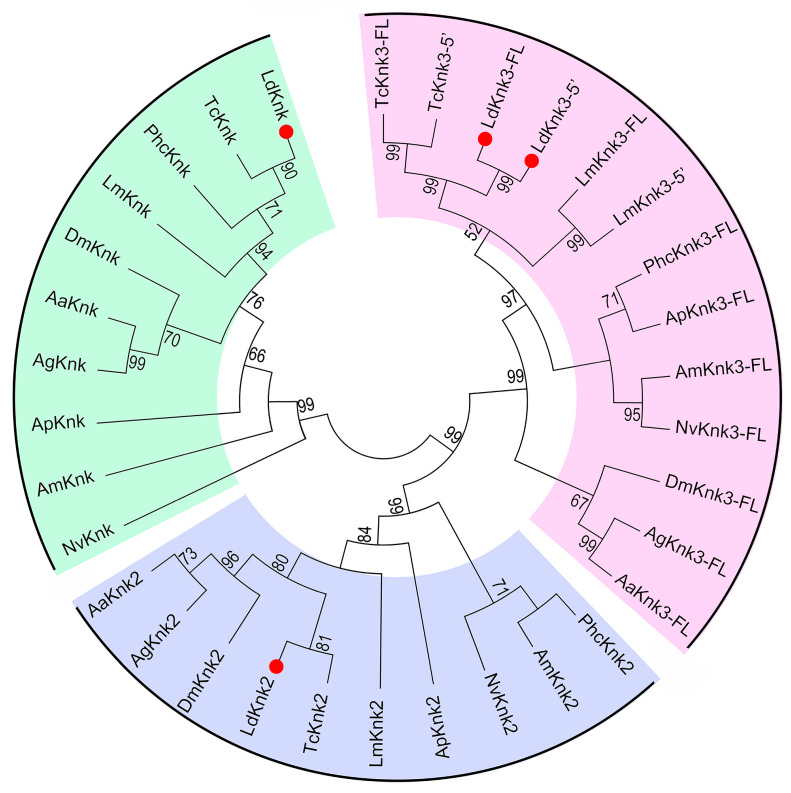
Phylogenetic tree of known insect Knk proteins. The unrooted phylogenetic trees were constructed using the neighbor-joining method with the Jones-TaylorThornton (JTT) substitution model, based on full-length protein sequence alignments. Bootstrap analyses with 1000 replications were performed, and bootstrap values >50% were shown on the tree. Knk proteins are derived from ten insect species: two Coleoptera *T. castaneum* (*Tc*) and *L. decemlineata* (*Ld*); an Orthoptera *L. migratoria* (*Lm*); an Anoplura *Pediculus humanus corporis* (*Phc*); a Hemiptera *Acyrthosiphon pisum* (*Ap*); two Hymenoptera *Apis mellifera* (*Am*) and *Nasonia vitripennis* (*Nv*); and three Diptera *D. melanogaster* (*Dm*), *Anopheles gambiae* (*Ag*) and *Aedes aegypti* (*Aa*). The accession numbers of the proteins are listed in Table S2.

A phylogenetic analysis was conducted to identify and classify the putative Knk-like proteins in *L. decemlineata*. This analysis involved aligning the amino acid sequences of Knk-like proteins from ten representative insect species. As a result, four Knk proteins were identified in *L. decemlineata* and were organized into three clades (Figure 2). *LdKnk* and *LdKnk2* belong to the Knk and Knk2 protein clades, respectively, while both *LdKnk3-FL* and *LdKnk3-5*′ belong to the Knk3 protein clade (Figure 2). This suggests the functional diversification of Knk proteins within *L. decemlineata*.

### 3.2. Spatio-Temporal Expression Patterns of LdKnk-Family Genes

**Figure 3:**
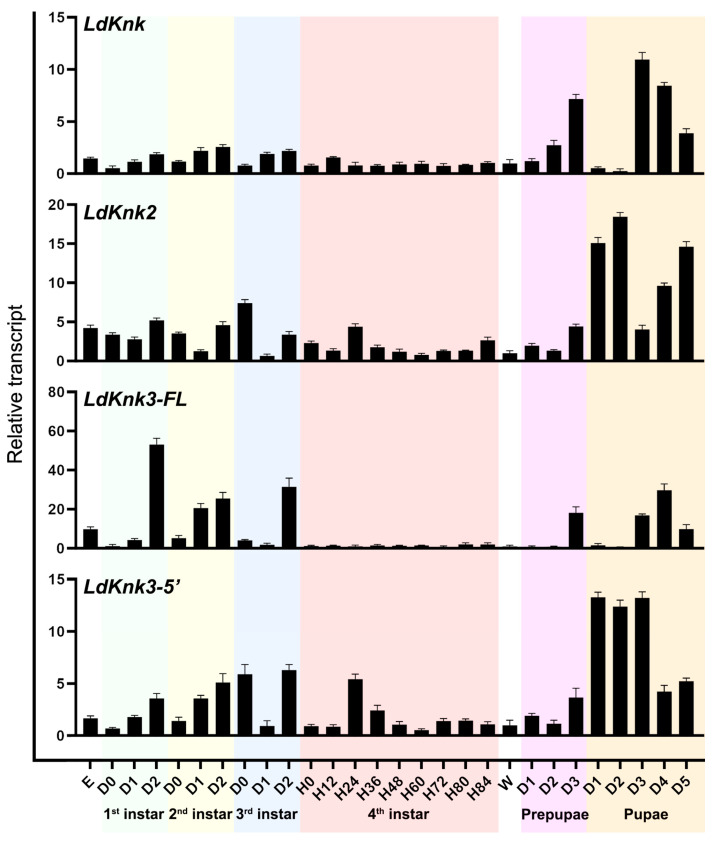
Temporal expression patterns of *LdKnk*-family genes during various developmental stages of *L. decemlineata*. The cDNA templates were prepared from pooled samples of mixed eggs (1–3 days old), first- to third-instar larvae, wandering larvae, prepupae, and pupae, collected daily. Additionally, fourth-instar larvae were sampled at 12-h intervals (D0/H0 indicated newly ecdysed larvae). Quantitative real-time PCR (qRT-PCR) was performed in triplicate, using technical replicates and three independent sample pools, each containing 5–10 individuals. Normalized expression values were determined using the 2^−ΔΔCt^ method, with the geometric mean of housekeeping genes for reference. Relative transcript levels are presented as ratios compared to wandering larvae (set to 1). Significant differences were analyzed using One-way ANOVA followed by the Tukey-Kramer test (*p* < 0.05) in Prism 10 software, with results listed in Table S3.

The expression patterns of *LdKnk*-family gene transcripts were analyzed at various developmental stages, including egg, larvae (first to fourth instar), wandering larvae, prepupae, and pupae, using qRT-PCR. The results indicate significant differences in gene expression (Figure 3). *LdKnk* exhibited relatively stable expression levels throughout the larval stages, with high expression at the end of the first to third instars and prepupal stages. A noticeable increase was observed during the prepupal and pupal stages, peaking in the mid-pupal stage (day 3). *LdKnk2* was expressed in all developmental stages, with its highest expression observed during the pupal stage. *LdKnk3-FL* displayed distinct expression patterns, with high transcript levels sharply increasing during the end stages of the first to third instars, prepupal, and mid-to-late pupal stages. Its expression was low or undetectable during other periods. *LdKnk3-5*′ exhibited elevated levels during the late stages of the first to third instars and the prepupal stage, with the highest expression observed from days 1 to 3 of the pupal stage. Unlike *LdKnk3-FL*, *LdKnk3-5*′ also exhibited high expression at the early third instar and 24 h into the fourth instar (Figure 3).

**Figure 4:**
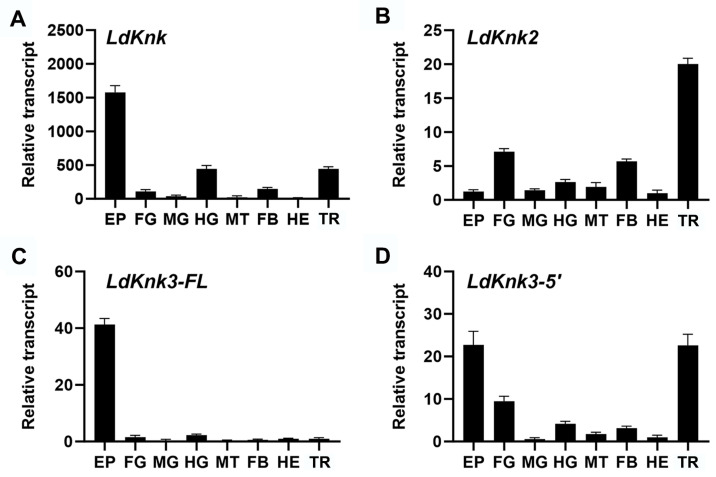
Tissue-specific expression profiles of *LdKnk*-family genes in fourth-instar larvae of *L. decemlineata* (**A**–**D**). The cDNA was obtained from epidermis (EP), foregut (FG), midgut (MG), hindgut (HG), malpighian tubules (MT), fat body (FB), hemocyte (HE), and tracheae (TR) of fourth-instar larvae. Tissue samples were organized into three independent pools, each containing 5–30 individuals, and analyzed in technical triplicates using qRT-PCR. Expression levels were quantified using the 2^−ΔΔCt^ method (±SE) and normalized to the geometric mean of housekeeping genes. The relative expression levels are presented with hemocytes set as 1. Significant differences were analyzed using one-way ANOVA followed by the Tukey-Kramer test (*p* < 0.05) in Prism 10 software, with results listed in Table S4.

The tissue-specific expression patterns of the *LdKnk*-family genes were analyzed in fourth-instar larvae. Relative transcript levels were measured in various tissues, including the epidermis (EP), foregut (FG), midgut (MG), hindgut (HG), Malpighian tubules (MT), fat body (FB), hemocytes (HE), and trachea (TR) (Figure 4). *LdKnk*, *LdKnk3-FL*, and *LdKnk3-5*′ showed predominant expression in the epidermis. However, *LdKnk2* was highly expressed in the trachea. Additionally, *LdKnk* and *LdKnk3-5*′ exhibited moderate expression in the trachea, and all four *LdKnk*-family genes were expressed in the digestive system, particularly in the foregut and/or hindgut. These findings suggest that the *LdKnk*-family genes play potential roles in cuticle formation and molting processes.

### 3.3. Effects of Silencing LdKnk Genes on Larval Development and Epidermis Structure

**Figure 5:**
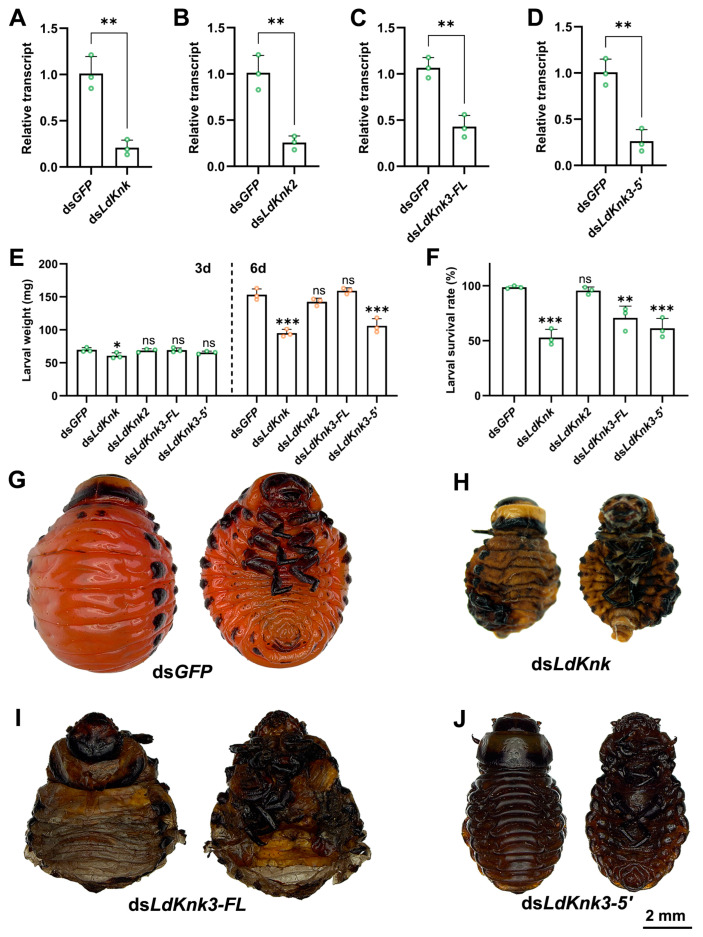
Effects of dietary ingestion of ds*LdKnk*-family genes on the second-instar larvae of *L. decemlineata*. Newly molted second-instar larvae were fed dsRNA-treated foliage for three days and then transferred to untreated leaves. Expression levels of *LdKnk*-family genes were analyzed on day 3 (**A**–**D**). Larval fresh weight was recorded on days 3 and 6 (**I**), and survival rates were recorded on day 6 (**J**). Data are displayed as bar charts with standard errors (±SE). Asterisks indicate significant differences (* *p* < 0.05, ** *p* < 0.01, *** *p* < 0.001). ds*GFP*-fed larvae reached the end of the fourth instar six days after the bioassay began (**E**). In contrast, *LdKnk*-family genes RNAi larvae displayed various abnormalities, including localized epidermal deformities, shrinkage, molting failure, and melanization, ultimately leading to mortality in some fourth-instar larvae (**F**–**H**).

RNAi experiments were conducted to investigate the biological functions of four *LdKnk*-family genes in *L. decemlineata* larvae. dsRNA specific to each *LdKnk*-family gene was produced using Escherichia coli HT115 and administered to newly molted second-instar larvae through ingestion of treated potato leaves. The larvae were fed dsRNA-soaked leaves for three days, followed by fresh potato leaves until pupation. ds*GFP* was used as the control. RNA was extracted from larvae three days after ingestion of dsRNA, and qRT-PCR was used to measure the transcript levels of the targeted genes. Results indicated significant gene silencing for the four *LdKnk*-family genes, evidenced by reduced transcript levels in treated larvae compared to controls (Figure 5A–D).

Silencing *LdKnk2* in larvae did not result in visible phenotypic changes. There were no significant differences in body weight or mortality between the *LdKnk2*-silenced group and the control group (Figure 5E,F). The larvae developed normally, molting and pupating successfully, similar to those in the control group (Figure 5G).

In contrast, knockdown of *LdKnk* caused significant larval weight reduction in larvae on the third and sixth days post-dsRNA feeding (Figure 5E). By the early fourth instar, about 50% of these larvae exhibited localized cuticle damage, and desiccation, leading to death (Figure 5F,H). Silencing *LdKnk3-5*′ also caused significant changes. on the sixth day post-treatment, these larvae had notable body weight reductions, and some exhibited melanization and death during the early fourth instar (Figure 5E,F,J).

Interestingly, the knockdown of *LdKnk3-FL* led to a slight increase in body weight on the sixth day, with no significant difference compared to controls (Figure 5E). However, the survival rate was significantly lower (Figure 5F). These larvae stopped developing and died on the soil surface before pupation, with the pupal cuticle trapped within the larval cuticle (Figure 5I). These results highlight the essential roles of *LdKnk*-family genes in the development and maintenance of the larval epidermis in *L. decemlineata*.

**Figure 6:**
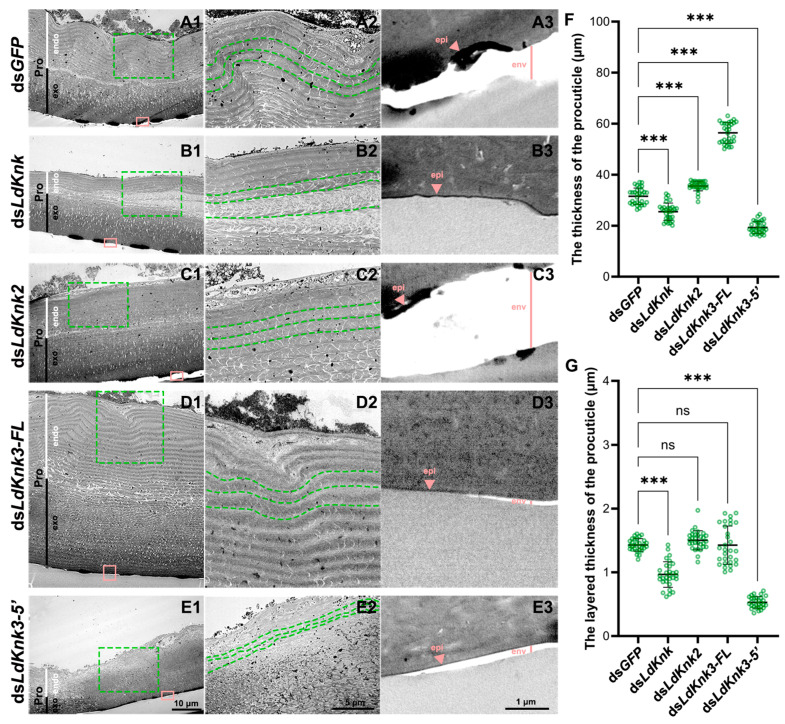
Impact of *LdKnk* genes Knockdown on Larval Cuticular Structure of *L. decemlineata*. TEM analysis of the epidermal structure in three-day-old fourth instar larvae following *LdKnks* knockdown. Panels (**A1**–**E1**) exhibit low-magnification views of the endocuticle and exocuticle within the procuticle (Pro). Panels (**A2**–**E2**) offer detailed views of the procuticle layer from the green boxes in A1–E1, with green dotted lines indicating the layer boundary. Panels (**A3**–**E3**) depict detailed views of the epicuticle and envelope cuticle from the orange boxes in A1–E1. Panels (**F**,**G**) present quantifications of overall and layered procuticle thickness, respectively, indicating significant (*** *p* < 0.001) and non-significant (ns) differences between the ds*GFP* control and treatment groups.

To investigate the roles of *LdKnk*-family genes in the structural organization of the larval epidermis, we conducted TEM analysis on the larval cuticle. In the control group treated with ds*GFP*-dsRNA, TEM revealed that the larval cuticle comprises three distinct layers: the outermost non-conductive transparent waxy envelope layer, the middle epicuticle, and the inner procuticle. The procuticle displayed a clear laminar structure, with a lighter-colored inner endocuticle (endo) and a darker-colored outer exocuticle (exo) (Figure 6(A1–A3)).

RNAi-mediated silencing of *LdKnk* resulted in significant thinning of the procuticle, including both the endocuticle and exocuticle, and disrupted the laminar organization, causing uneven thickness and localized expansion (Figure 6(B1,B2),F,G vs. Figure S1A,B). Silencing *LdKnk3-5*′ caused the larval epidermis to become thinner, with both the procuticle and its endocuticle and exocuticle significantly reduced in thickness (Figure 6(E1,E2),F,G vs. Figure S1A,B). The chitin layers were also markedly thinner, with a blurred layer structure (Figure 6(D2)). In contrast, silencing *LdKnk3-FL* led to a significantly thicker larval procuticle, nearly twice that of the control, with both the endocuticle and exocuticle substantially thicker, while the chitin layer thickness remained similar to the control (Figure 6(D1,D2),F,G vs. Figure S1A,B). Knockdown of *LdKnk2* resulted in larvae with procuticle morphology and thickness similar to the control group, with only an increase in procuticle thickness due to a thicker exocuticle (Figure 6(B1,B2),F,G vs. Figure S1A,B).

Measurements of the epicuticle and envcuticle thickness revealed that *LdKnk2* knockdown significantly increased the thickness of both layers compared to the control group (Figure 6(B3) vs. Figure S1C,D). In contrast, knockdowns of *LdKnk*, *LdKnk3-FL*, and *LdKnk3-5*′ resulted in significantly thinner epicuticle and envcuticle layers (Figure 6(B3–E3) vs. Figure S1C,D). These findings suggest that *LdKnk*-family genes play distinct roles in maintaining the structural integrity of the larval cuticle.

## 4. Discussion

*Knickkopf* genes are essential for the formation and maintenance of insect cuticles, particularly in organizing and protecting chitin within the exoskeleton [10]. However, research on *Knk* genes has primarily focused on a few model species, such as *D. melanogaster* [9,23,26], *T. castaneum* [28,29,30], and *L. migratoria* [22,31,32], leaving their broader roles in insects largely unexplored. In this study, we identified and characterized *Knk* genes in *L. decemlineata*, a globally destructive pest of potatoes. Our findings reveal the distinct and essential functions of *LdKnk*-family genes in the larval cuticle, thereby enhancing our understanding of the molecular mechanisms underlying cuticle formation and maintenance and elucidating the developmental roles these genes play.

In our study, we identified and characterized four *LdKnk*-family coding gene transcripts in the genome and transcriptome of *L. decemlineata*. Phylogenetic analysis revealed that the *Ld*Knk-family proteins are divided into three distinct clades: Knk, Knk2, and Knk3. We found single transcripts for *LdKnk* and *LdKnk2* genes, whereas *LdKnk3* gave rise to two alternatively spliced transcripts, coding for a full-length protein (*LdKnk3-FL*) and a C-terminally truncated version (*LdKnk3-5*′). This pattern in the *L. decemlineata* is somewhat similar to that in *T. castaneum*, *D. melanogaster*, and the hemimetabolous insect *L. migratoria*. Domain analyses showed that *Ld*Knk, *Ld*Knk2, and *Ld*Knk3-FL each consist of a signal peptide, two tandem DM13 domains, and a DOMON domain, whereas *Ld*Knk3-5′ lacks one DOMON domain, which is essential for Knk function in *D. melanogaster* [25]. These findings suggest that, despite the presence of three *Knk* coding genes across different insect species, there may be functional differences among them.

To further investigate the function of *LdKnk*-family genes in *L. decemlineata*, we used qRT-PCR to analyze the expression of the four *LdKnk*-family genes at different developmental stages and in various tissues. The results showed that all four *LdKnk*-family genes were consistently highly expressed before larval molting, at the late prepupal stage, and throughout the pupal stages, with expression patterns closely mirroring those of 20-hydroxyecdysone (20E). In terms of tissue expression, with the exception of *LdKnk2*, which was predominantly expressed in the trachea, the other *LdKnk*-family genes were significantly expressed in the larval epidermis (Figure 3 vs. Figure 4). Similarly, in *L. migratoria* and *T. castaneum*, *Knk* genes (*LmKnk*, *LmKnk3-FL*, *LmKnk3-5*′, *TcKnk*, *TcKnk2*, and *TcKnk3-FL*) showed continuous and high expression in late instar larvae, particularly in the epidermis, with *LmKnk*-family genes in *L. migratoria* confirmed as downstream targets of 20E [28,31,32]. These patterns indicate that insect *Knk* genes are significantly upregulated during cuticle formation and molting processes, highlighting their crucial roles in these stages.

The insect protein Knk is conserved across various insect species and plays a crucial role in cuticle formation and maintenance. Our RNAi experiments demonstrate that silencing *LdKnk* significantly impacts larval development and cuticle integrity. Knockdown of *LdKnk* in larvae results in significant weight loss, with more than half dying before the prepupal stage. Most larvae exhibit localized epidermal damage, melanization, dehydration, and death. TEM analysis supports these observations, showing that silencing *LdKnk* significantly reduces the thickness of the procuticle and its layers, leading to thinner epicuticle and endocuticle layers and compromised cuticle integrity (Figure 5 and Figure 6). Similarly, in *L. migratoria*, *LmKnk* localizes to the chitin layer of the cuticle. Injecting ds*LmKnk* reduces chitin content, disrupts crystalline chitin organization, and lowers lipid content, impairing the cuticle’s barrier function [31]. Previous research in *D. melanogaster* shows that *DmKnk* is crucial for cuticle formation and epithelial tube morphogenesis [24,25]. RNAi knockdown results in malformed wings and an amorphous procuticle, while overexpression maintains normal structure [23]. *Dm*Knk, along with *Dm*kkv and *Dm*cystic, is essential for organizing chitin in tracheal tubes. Rescue experiments confirm that proper *Dmknk* expression restores tube integrity [9,45]. In *T. castaneum*, *Tc*Knk proteins protect newly synthesized chitin from degradation and ensure proper laminar organization. RNAi for *TcKnk* causes developmental arrest at each molt, highlighting its role in chitin stability [28]. The Retroactive (Rtv) protein is essential for trafficking *Tc*Knk into the procuticle. *Tc*Rtv localizes within epidermal cells and promotes *Tc*Knk movement, protecting the new cuticle from chitinases. Loss of *TcRtv* leads to *TcKnk* entrapment and molting defects [7,29,30]. The conserved function of Knk protein across insects underscores its essential role in chitin organization and cuticle integrity.

Knk3 exhibits multiple alternative splicing variants that vary across species. *LdKnk3* has two transcripts, similar to *LmKnk3*, while *TcKnk3* produces three [28,32]. In *L. decemlineata*, the roles of *LdKnk3-FL* and *LdKnk3-5*′ are particularly notable due to their opposing effects on cuticle thickness. Silencing *LdKnk3-FL* results in a thicker cuticle, leading to molting disruption before the prepupal stage, while silencing *LdKnk3-5*′ results in a thinner cuticle, causing melanization and death (Figure 5 vs. Figure 6). This suggests that proper procuticle formation is crucial for insect molting and development. Similarly, in *L. migratoria*, silencing *LmKnk3-5*′ causes molting defects and high mortality, significantly decreasing chitin content without affecting cuticle structure and impeding lipid deposition on the cuticle surface [32]. In *T. castaneum*, dsRNA targeting multiple exons of *TcKnk3* had varied effects. Injection of exon 9-specific dsRNA, which depletes *TcKnk3-FL* transcripts, caused 100% mortality at the pharate adult stage. Silencing *TcKnk3-3*′ specifically led to molting arrest at the same stage. Knocking down all three *TcKnk*-family genes significantly reduced total chitin content to varying degrees. *TcKnk3* transcripts are crucial for the integrity of body wall denticles and tracheal taenidia but are not essential for the elytral and body wall procuticles [28]. These findings highlight the complex roles of Knk3 isoforms in different insect species and their critical functions in maintaining cuticle structure and integrity.

In this study, silencing *LdKnk2* in larvae did not result in visible phenotypic changes. However, measurements revealed that *LdKnk2* knockdown significantly increased the thickness of the epicuticle and endocuticle compared to the control group. Similarly, in *L. migratoria*, no visible phenotypic changes were observed after *LmKnk2* was silenced by RNAi [32]. In contrast, silencing *TcKnk2* in *T. castaneum* caused molting arrest at the pharate adult stage, indicating that *TcKnk2* is crucial for the integrity of body wall denticles and tracheal taenidia, but not for the elytral and body wall procuticles [28].

Understanding the specific roles of *LdKnk*-family genes in *L. decemlineata* provides valuable insights for developing targeted pest management strategies. The essential functions of *LdKnk*, *LdKnk3-FL*, and *LdKnk3-5*′ in maintaining cuticle integrity make them promising targets for RNAi-based pest control. For instance, silencing *LmKnk3-5*′ in *L. migratoria* not only reduced procuticle thickness but also increased susceptibility to four insecticides from three different classes. Disrupting these *LdKnk*-family genes could therefore weaken the protective cuticle barrier, making insects more vulnerable to environmental stressors and enhancing insecticide efficacy. Our findings on the opposite effects of *LdKnk3-FL* and *LdKnk3-5*′ on cuticle thickness further underscore the importance of precise regulation of the cuticle in pest control applications. This research enhances our understanding of insect cuticle biology and offers new genetic insights for improving pest management strategies.

## 5. Conclusions

Our study has successfully identified and analyzed four *LdKnk*-family genes in the larval epidermis of *L. decemlineata*. The genes *LdKnk*, *LdKnk2*, *LdKnk3-FL*, and *LdKnk3-5*′ are integral to cuticle formation and maintenance. RNAi experiments revealed that silencing these genes results in significant changes in cuticle structure, larval development, and survival rates. Specifically, *LdKnk* and *LdKnk3-5*′ are essential for maintaining cuticle integrity and preventing damage, while *LdKnk3-FL* regulates cuticle thickness, ensuring proper molting. These findings suggest that targeting *LdKn*k-family genes could be an effective strategy for developing RNAi-based pest control methods, potentially reducing the impact of the beetle on potato crops. Future research should further clarify these genes’ roles and optimize RNAi techniques for practical agricultural use.

## Figures and Tables

**Table 1:** Characteristics of four *LdKnk*-family genes in the *L. decemlineata*.

Gene Name	Accession Number	cDNA (bp)	Coding Region	5′-UTR (bp)	3′-UTR (bp)	aa	pl	Mw(kDa)
*LdKnk*	PP962378	2118	61–2088	60	30	675	6.35	76.03
*LdKnk2*	PP962379	2142	1–2109	0	33	702	5.09	78.90
*LdKnk3-FL*	PP962380	5100	49–4236	48	864	1395	7.41	156.48
*LdKnk3-5*′	PP962381	1042	61–975	60	67	304	7.65	33.81

UTR–untranslated region; aa–amino acid; pl–isoelectric point; Mw–molecular weight.

## Data Availability

All data generated in association with this study have been made available in the Supplementary Materials published online with this article.

## Supplementary Materials

The following supporting information can be downloaded at: www.mdpi.com/article//s1, Figure S1. Cuticle thickness in fourth-instar *L. decemlineata* larvae following RNAi of *LdKnk*-family genes. Table S1. Primers used in RT-PCR, RACE, ORF verification, dsRNA synthesis, and qRT-PCR. Table S2. Names and accession numbers of the proteins used for phylogenetic tree construction. Table S3. Significance analysis of *LdKnk-family* gene expression across developmental stages using one-way ANOVA and Tukey-Kramer test. Table S4. Significance analysis of *LdKnk-family* gene across tissues using one-way ANOVA and Tukey-Kramer test.
