# Supplementary material for: Identification and Functional Insights of Knickkopf Genes in the Larval Cuticle of Leptinotarsa decemlineata
Source: Insects. 2024 Aug 19;15(8):623. doi: 10.3390/insects15080623 (PMC11354708; doi:10.3390/insects15080623)
Supplement: Supplementary file 1 [file insects-15-00623-s001.zip › insects-3157294 Supplementary 1.pdf]

Supplementary data

Identification and Functional Insights of *Knickkopf* Genes in the Larval Cuticle of *Leptinotarsa decemlineata*

Mu-Zi Zeng[1,†], Wei Zhou[1,†], Shan-Shan Wen[1,†], Hao Wu[1], Qing Zhang[1], Kai-Yun Fu[2], Wen-Chao Guo[2], Ji-Feng Shi[1,*]

1 State Key Laboratory of Resource Insects; College of Sericulture, Textile, and Biomass Sciences, Southwest University, Chongqing, 400715, China; MZ_Zeng@163.com (M.-Z.Z.); zorroice@yeah.net (W.Z.); wenshanshan@whu.edu.cn (S.-S.W.); wuhao2944@163.com (H.W.); zhq20190810@swu.edu.cn (Q.Z.)

2 Institute of Plant Protection Xinjiang Academy of Agricultural Sciences/Key Laboratory of Integrated Pest Management on Crops in Northwestern Oasis, Ministry of Agriculture/Xinjiang Key Laboratory of Agricul-tural Biosafety, Urumqi 830091, China; fukaiyun000@foxmail.com (K.-Y.F.); gwc1966@163.com (W.-C.G.)

* Correspondence: shijifeng@swu.edu.cn; Tel.: +86-23-68251035

† These authors contributed equally to this work.

Table of Contents:

Figure S1. Cuticle thickness in fourth-instar *L. decemlineata* larvae following RNAi of *LdKnk*-family genes.

Table S1. Primers used in RT-PCR, RACE, ORF verification, dsRNA synthesis, and qRT-PCR.

Table S2. Names and accession numbers of the proteins used for phylogenetic tree construction.

Supplementary Figure S1

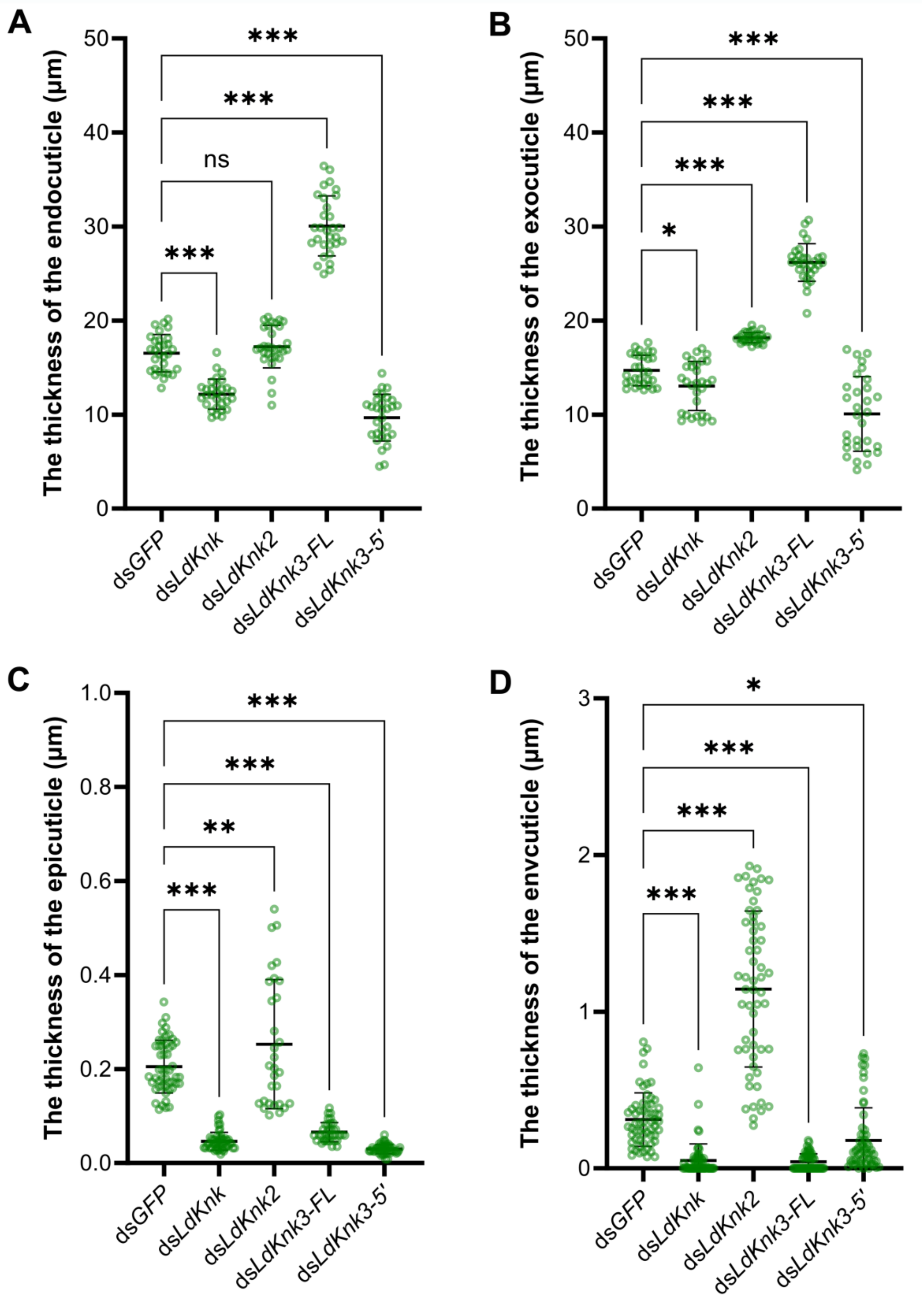

Figure S1. Cuticle thickness in fourth-instar *L. decemlineata* larvae following RNAi of *LdKnk*-family genes. Early second-instar larvae were fed ds*GFP* or ds*LdKnk*-family genes, and the cuticle layers were examined on the third day of the fourth instar. Measurements include (A) the Thickness of the endocuticle, (B) the Thickness of the exocuticle, (C) the thickness of the epicuticle, and (D) the thickness of the envelope cuticle. Significant differences between the ds*GFP* control and treatment groups are indicated as * p<0.05, ** p<0.01, *** p<0.001, ns: non-significant.

Supplementary Tables

Table S1. Primers used in RT-PCR, RACE, ORF verification, dsRNA synthesis, and qRT-PCR.

Fragment name	Forward primer	Reverse primer
**RT-PCR**		
LdKnk-1	GCCAGCATTTCACGATTT	CACAGTGCCAGCCTCATA
LdKnk-2	ACTGCTCCTAAATTCCTTC	ATCAGCGACCTCCTGTAC
LdKnk2-1	CAGTATTACGGGCTTACA	CTTACCTATGATCCTAGCAG
LdKnk2-2	AAGGGGAGGTTAGTGTCA	ATCGTCAGAAATCGTAAAG
LdKnk3-FL-1	CAATACTTGGGCAAACTC	CCTCCTCTTCCACCTTCC
LdKnk3-FL-2	AGGAGGTATCATTCCACA	TTGGCTTTACCCTTGTTT
LdKnk3-FL-3	CGGTTCAGACTTAGACGA	GAACTTCCGTTGGTAGAG
LdKnk3-5'	AAAGCCATAGTGCTCAAT	CACAACACTGGATGAAAC
**RACE**		
LdKnk2 5'-GSP		TGCCAGCCAGTCTATGTCGTAA
LdKnk2 5'-NGSP		CCCTCGGTTTGGGGACTTCTA
LdKnk3-FL 5'-GSP		GTGACGCCTTCCTTTCGGTA
LdKnk3-FL 5'-NGSP		TGGGTGCTGGCGTAGAAATAG
LdKnk3-FL 3'-GSP	AAGAAAGTGTCGGGGCAGAGC	
LdKnk3-FL 5'-NGSP	TAGGGTTCTCAGCAGGGATTTG	
**ORF verification**		
LdKnk	TTCTTTGTTGTACTGTTTGCCA	TCACAGTGCCAGCCTCAT
LdKnk2	GGTAGATAAGGAGAAAGCG	TCAGAAATCGTAAAGCTC
LdKnk3-FL	CAATACTTGGGCAAACTC	CTTACAGAATCAGTAGCACC
LdKnk3-5'	ATGGCCAGGATCGGGCCCATC	ACAACACTGGATGAAACG
**dsRNA synthesis**		
ds*Knk*-1	GCCCAACCTGCTTCCAAAG	TGTTATAGCGGGACCGAAAATC
ds*Knk*-2	TTGGGCCAGTATAAAGGGGTGT	TCGGGCTTCGATCTTGTGAA
ds*Knk2*-1	GCAATCCAACAAGCAGCACC	TCCGTCGCACTGAGCCAT
ds*Knk2*-1	ATCAACGATCAGCCAATCCCC	CCCTCCTCATCCGACAACTCC
ds*Knk3-FL*-1	CACCGCGTCCTTCGCTTTA	TGGGCAGACACCTCAACATTTC
ds*Knk3-FL*-2	CCCGAAGTCTCAGCAAAGTACC	TGTCTGCGTCAGGCGTCC
ds*Knk3-5'*-1	TACTATGGACAACTTATGG	CACAACACTGGATGAAAC
ds*Knk3-5'*-2	AGCCCACAGTGTTCCTAC	ATGAGCAATGAAAGCATT
ds*egfp*	AAGTTCAGCGTGTCCG	CACCTTGATGCCGTTC
**qRT-PCR**		
q*LdKnk*	TCGTACATCGGACCTTCTGG	GGAGCTAGGAGGCCATTGAT
q*Ld Knk2*	GGTGTGGACTACGACCAAGA	CTCCAGCCTTAGGGTTTCCA
q*Ld Knk3-FL*	TGGTCGTCTTTGTCACTGGA	TCGGTGAGTGAAGCACTGAT
q*Ld Knk3-5'*	CACGTGCTCATCCCTCAAAG	TCACCCATACACAACACTGGA
q*LdRP4*	AAAGAAACGAGCATTGCCCTTCCG	TTGTCGCTGACACTGTAGGGTTGA

q*LdRP18*	TAGAATCCTCAAAGCAGGTGGCGA	AGCTGGACCAAAGTGTTTCACTGC
q*LdARF1*	CGGTGCTGGTAAAACGACAA	TGACCTCCCAAATCCCAAAC
q*LdARF4*	GTGCTCGTGAACCATGTGAA	AACCTCCAATCCCTCGTGAA

Table S2. Names and accession numbers of the proteins used for phylogenetic tree construction

Species	Homologous proteins	Accession number	Order
Drosophila melanogaster	*Dm*Knk	NP_649981.1	Diptera
	*Dm*Knk2	NP_001097889.2	
	DmKnk3-FL	NP_001027171.1	
Anopheles gambiae	*Ag*Knk	XP_313797.4	
	*Ag*Knk2	XP_308115.4	
	*Ag*Knk3-FL	XP_313250.2	
Aedes aegypti	*Aa*Knk	XP_001657332.1	
	*Aa*Knk2	XP_001657415.1	
	*Aa*Knk3-FL	XP_001657668.1	
Pediculus humanus corporis	*Phc*Knk	XP_002430062.1	Anoplura
	*Phc*Knk2	XP_002429468.1	
	*Phc*Knk3-FL	XP_002425509.1	
Acyrthosiphon pisum	*Ap*Knk	XP_001950825.2	Hemiptera
	*Ap*Knk2	XP_001946688.2	
	*Ap*Knk3-FL	XP_003241759.1	
Apis mellifera	*Am*Knk	XP_394084.4	Hymenoptera
	*Am*Knk2	XP_393508.4	
	*Am*Knk3-FL	XP_003250319.1	
Nasonia vitripennis	*Nv*Knk	XP_001606352.1	
	*Nv*Knk2	XP_001602771.1	
	*Nv*Knk3-FL	XP_001606495.2	
Tribolium castaneum	*Tc*Knk	AEM60136.1	Coleoptera
	*Tc*Knk2	AHA05987.1	
	*Tc*Knk3-FL	XP_968712.2	
	*Tc*Knk3-5’	AHA05990.1	
Locusta migratoria	*Lm*Knk	ANA57447.1	Orthoptera
	*Lm*Knk2	MT080984	
	*Lm*Knk3-FL	MT080985	
	*Lm*Knk3-5’	MT080986	
